# Triggered Migraine Attack by Flickering Fluorescent Lights in an Assembly Line Worker: A Case Report

**DOI:** 10.7759/cureus.95794

**Published:** 2025-10-31

**Authors:** Albert Gassull

**Affiliations:** 1 Emergency Medicine, Hospital Sant Joan de Reus, Tarragona, ESP

**Keywords:** case report, environmental trigger, factory worker, flickering lights, headache, known disease, migraine, occupational health, reversible, transient symptoms

## Abstract

Migraine is a prevalent neurological disorder often exacerbated by environmental triggers, including visual stimuli like flickering lights, posing unique challenges in occupational settings such as factories. We describe a 32-year-old male assembly line worker with a history of episodic migraine without aura who experienced a sudden-onset unilateral headache, nausea, and photophobia while exposed to flickering fluorescent overhead lighting during a routine shift. He reported no recent alcohol or caffeine overuse, or medication changes, and denied trauma or infections. Clinical examination was unremarkable, with no neurological deficits, fever, or meningeal signs. The episode resolved completely within four hours after relocation to a quiet, darkened break area for rest, without pharmacological intervention. No diagnostic tests or imaging were pursued, given his established migraine diagnosis and clear temporal link to the trigger. Follow-up at one and three months confirmed no recurrence with workplace accommodations, including light filters and shift adjustments. This case highlights the role of artificial lighting as a preventable occupational trigger for migraine, emphasizing the need for environmental assessments in industrial environments to reduce absenteeism and improve worker well-being. Early recognition and simple modifications can avert acute attacks, underscoring occupational health's importance in managing chronic conditions like migraine.

## Introduction

Migraine affects approximately 15% of the global population, with significant socioeconomic impact through lost productivity, particularly in manual labor sectors where environmental factors can precipitate attacks [1]. Common triggers include stress, hormonal fluctuations, dietary factors, and sensory stimuli, but visual triggers, such as bright or flickering lights, are reported by up to 50% of migraineurs, often leading to photophobia and headache exacerbation [2]. In occupational contexts, assembly line workers face prolonged exposure to artificial lighting, noise, and repetitive tasks, heightening vulnerability to such triggers and potentially classifying migraine as a work-aggravated condition.

Flickering fluorescent lights, common in factories due to cost-effectiveness, emit imperceptible high-frequency oscillations that can disrupt cortical processing in susceptible individuals, mimicking patterns known to induce visual discomfort and migraines [3]. Studies suggest that migraineurs exhibit altered visual evoked potentials and reduced habituation to repetitive stimuli, explaining heightened sensitivity to flicker [4]. Comorbidities like anxiety or sleep disruption can amplify risks, but isolated triggers offer opportunities for targeted prevention.

This report details an acute migraine episode directly linked to workplace lighting, an underappreciated factor in occupational health. By illustrating rapid resolution through environmental modification, it advocates for proactive interventions like light audits and ergonomic adjustments to mitigate disability. Such cases inform policy, as untreated triggers contribute to chronicity and healthcare costs.

## Case presentation

A 32-year-old assembly line worker presented to our occupational health clinic complaining of a sudden-onset right-sided throbbing headache, accompanied by nausea and sensitivity to light, which began midway through his morning shift. Employed in electronics manufacturing for eight years, he had a documented history of episodic migraine without aura (per the International Classification of Headache Disorders 3 (ICHD-3) criteria) since age 25, occurring three to four times annually and typically managed with over-the-counter ibuprofen as needed. He described the current episode as similar to prior migraines but noted its abrupt start after repositioning under a bank of overhead fluorescent lights that he perceived as "flickering more than usual" due to recent maintenance issues. Symptoms escalated within 15 minutes, forcing him to halt work; he denied visual aura, vomiting, vertigo, or focal weakness. No recent stressors, sleep deprivation, dietary indiscretions (e.g., skipped meals, chocolate, and cheese), alcohol consumption, or caffeine overuse were reported. His medications were limited to occasional paracetamol, with no changes in the preceding weeks. Family history included maternal migraines, but he had no personal comorbidities like hypertension, allergies, or psychiatric conditions.

Upon arrival at the clinic 30 minutes post-onset, the patient appeared distressed but alert and oriented. Vital signs were normal (blood pressure = 124/78 mmHg, heart rate = 88 bpm, temperature = 36.8°C, respiratory rate = 16/min). Neurological examination revealed no cranial nerve abnormalities, intact motor and sensory function, normal reflexes (2+ symmetric) [5], and a negative Romberg test [6]. Fundoscopy was unremarkable without papilledema (Table 1) [7]. There were no meningeal signs (negative Kernig and Brudzinski signs) [8], and the neck was supple. Given his established migraine diagnosis and absence of red flags (e.g., thunderclap onset, fever, altered consciousness, or new neurological deficits) [9], no laboratory investigations, neuroimaging, or EEG were deemed necessary.

**Table 1:** Key negative findings. TIA: transient ischemic attack; SAH: subarachnoid hemorrhage; BP: blood pressure; mmHg: millimeters of mercury; °C: degrees Celsius.

Assessment	Result	Clinical significance
Neurological exam	No focal deficits, normal reflexes (2+), negative Romberg	Rules out stroke/TIA, structural lesions
Meningeal signs	Negative Kernig/Brudzinski, supple neck	Excludes meningitis/SAH
Fundoscopy	No papilledema	No increased intracranial pressure
Vital signs	BP 124/78 mmHg, afebrile (36.8°C)	No hypertensive crisis or infection
Associated symptoms	No thunderclap onset, fever, or altered consciousness	Supports primary migraine diagnosis
History	No recent medication changes, substance use, trauma	Eliminates secondary causes

Management focused on trigger removal. He was immediately relocated to a dimly lit, quiet break room with no fluorescent exposure, provided hydration, and advised to rest supine with eyes closed. No acute medications were administered, per his preference and the episode's moderate severity (rated 6/10 on the Visual Analog Scale (VAS)).

**Figure 1:**
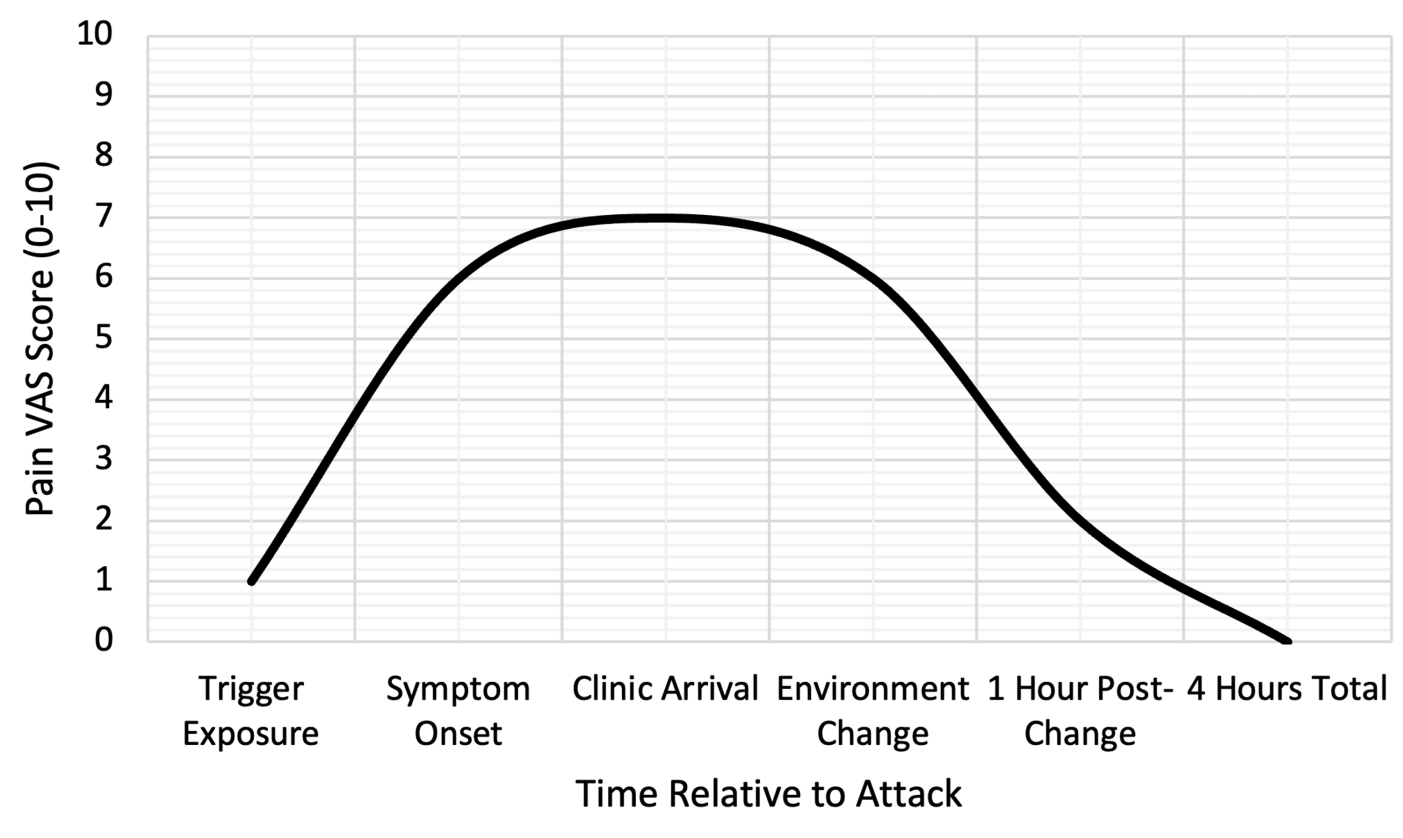
Migraine attack resolution curve. VAS: Visual Analog Scale.

To quantify the attack's trajectory and guide management, the patient was asked to score his headache intensity orally using a 0-10 VAS [10], where 0 represents no pain and 10 the worst imaginable pain, reporting moderate severity at 6/10 upon clinic arrival, escalating briefly to peak discomfort before environmental relocation. This subjective assessment, tracked verbally during the observation period, documented initial stabilization within one hour of trigger removal, followed by a steady decline to complete resolution by four hours post-onset (Figure 1). This resolution timeline aligns with typical migraine recovery patterns in triggered episodes, where removal of the precipitant often leads to faster symptom abatement compared to untreated attacks, which may persist for four to 72 hours per the ICHD-3 guidelines. The pain trajectory illustrates the rapid efficacy of non-pharmacologic intervention, i.e., relocation to a flicker-free, darkened environment, without acute medications, aligning with his preference and the episode's characteristics. Such real-time monitoring reinforced diagnostic confidence in a triggered migraine exacerbation rather than progression to status migrainosus or secondary pathology.

At one-week review, he reported no residual effects and resumed work with temporary accommodations, including assignment to stations with stable light-emitting diode (LED) lighting and permission for breaks in low-light areas. Occupational safety assessed the implicated lights, confirming flicker from aging ballasts. At one- and three-month follow-ups, no further attacks occurred, attributed to implemented changes like light filters and ergonomic evaluations. He maintained good compliance with lifestyle advice, reporting improved overall migraine control.

## Discussion

This case exemplifies an acute migraine attack precipitated by flickering fluorescent lights in an occupational setting, a trigger supported by evidence of heightened visual sensitivity in migraineurs [1]. Assembly line environments, with constant artificial illumination, amplify such risks, as flicker disrupts cortical adaptation and exacerbates trigeminovascular activation [2]. Our patient's rapid symptom onset under faulty lights aligns with reports of photic hypersensitivity, where even imperceptible high-frequency oscillations (e.g., 100-120 Hz) can induce discomfort and attacks, particularly in those with pre-existing migraine [3].

The absence of aura and resolution without medication highlight a classic triggered episode, distinguishable from tension-type headache (TTH) by unilateral throbbing and associated nausea/photophobia [4]. Occupational health's role was pivotal in facilitating prompt interventions. Literature indicates that fluorescent lamps, emitting uneven spectra and flicker, are common culprits for visual stress, with studies showing reduced migraine frequency through spectral filtering or LED replacements [11]. Neural mechanisms involve intrinsically photosensitive retinal ganglion cells (ipRGC)-mediated pathways modulating thalamic nociceptive neurons, explaining light's pain-amplifying effect even in dim conditions [12].

Diagnostic restraint here, i.e., no labs or imaging, was appropriate, per guidelines for known migraine without alarms, avoiding unnecessary costs and radiation [13]. However, in atypical cases, exclusion of mimics like cluster headache or secondary causes (e.g., glaucoma and sinusitis) is essential. This report's novelty lies in the workplace context: factory workers endure prolonged exposure, with flicker from aging fixtures potentially qualifying as an occupational hazard under health regulations. Meta-analyses confirm visual stimuli as a top perceived trigger (32% endorsement), more so in migraine than TTH, underscoring environmental modifications' preventive value [14].

Therapeutic implications favor non-pharmacologic strategies; our patient's swift recovery with rest and trigger avoidance mirrors trial outcomes where light modulation halves attack rates. Limitations include reliance on self-report and lack of polysomnography or evoked potentials, which could quantify flicker sensitivity. Future occupational protocols should incorporate routine lighting assessments, education on triggers, and accommodations like tinted glasses or breaks, reducing absenteeism estimated at 1.3 billion workdays annually from migraine. Broader research on industrial ergonomics could validate causality, informing policies to mitigate this underrecognized burden.

## Conclusions

This case illustrates how flickering fluorescent lights can acutely trigger migraine in susceptible workers, resolved simply by environmental adjustment. Occupational health interventions, like light modifications, offer effective prevention without drugs. Greater awareness and workplace reforms are key to minimizing such impacts on productivity and quality of life.
